# Metabolic risk factors for fungal ball sinusitis in perimenopausal women

**DOI:** 10.3389/falgy.2026.1900638

**Published:** 2026-10-19

**Authors:** Shi-Yun Wang, Wei Xu, Yue-Xi Liu, Liang Jiang, Yong Xu, Fang-Yuan Teng, Xiao-Yan Hu, Hong-Xia Li

**Affiliations:** 1 Department of Otolaryngology-Head and Neck Surgery, The Affiliated Hospital of Southwest Medical University, Luzhou, Sichuan, China; 2 Metabolic Vascular Diseases Key Laboratory of Sichuan Province, and Metabolic Vascular Diseases Key Laboratory of Sichuan-Chongqing Cooperation, Luzhou, Sichuan, China; 3 Department of Endocrinology and Metabolism, The Affiliated Hospital of Southwest Medical University, Luzhou, Sichuan, China; 4 Department of Pathogen Biology, School of Basic Medicine, Southwest Medical University, Luzhou, Sichuan, China; 5 Public Center of Experimental Technology of Pathogen Biology Technology Platform, Southwest Medical University, Luzhou, Sichuan, China

**Keywords:** chronic rhinosinusitis, fungal ball sinusitis, HDL-C, perimenopausal women, TyG-BMI

## Abstract

**Objectives:**

Rising incidence of fungal ball sinusitis (FBS) is frequently observed in perimenopausal women. This study aimed to explore the relationships between TyG-BMI, HDL-C and FBS in this specific population.

**Study design:**

This single-center retrospective study enrolled FBS patients from 2015 to 2025. Perimenopausal women aged 45–60 years were selected as the study subjects, and patients with chronic non-fungal sinusitis (CNFS) were selected as the control group. Biochemical indicators were compared, and participants were stratified by TyG-BMI quartiles. Logistic regression was used to identify independent risk factors, and ROC curves were applied to assess predictive efficiency.

**Results:**

The cases of FBS showed an increasing trend. The patients were predominantly middle-aged and elderly individuals, and the proportion of female patients was significantly higher than that of males (63.96% vs. 36.04%), with the peak age of female morbidity concentrated between 45 and 60 years. Intergroup comparison demonstrated that the FBS group had significantly lower HDL-C levels and significantly higher TyG and TyG-BMI values than the CNFS group. With the elevation of TyG-BMI quartiles, the HDL-C level decreased progressively, accompanied by a significantly rising trend in the prevalence of FBS (*P* < 0.05). Logistic regression analysis confirmed that HDL-C and quartile of TyG-BMI were independent influence factors for FBS in perimenopausal women (*P* = 0.008, OR = 0.482,95%CI:0.282–0.823; *P* = 0.04, OR = 1.192, 95%CI:1.008–1.409; respectively). ROC analysis revealed that the combined model of TyG-BMI and HDL-C exhibited limited predictive value for FBS.

**Conclusion:**

The incidence of FBS has been increasing annually, especially in perimenopausal women. Decreased HDL-C and elevated TyG-BMI are independent metabolic risk factors for FBS in perimenopausal women. Targeted metabolic monitoring and early intervention may be associated with lower FBS risk.

## Graphical Abstract

**Figure:**
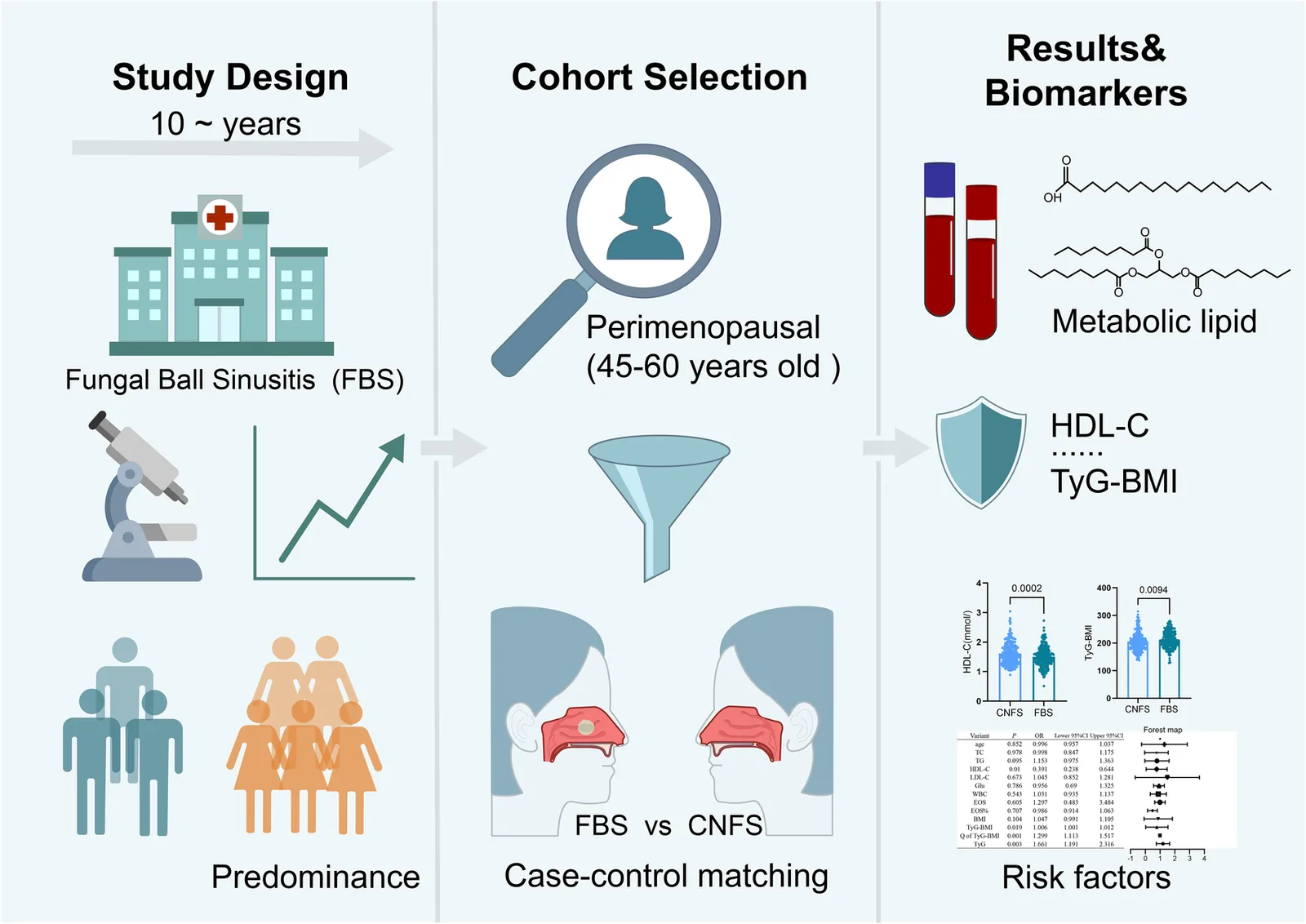

## 1 Introduction

Fungal ball sinusitis (FBS) is a common chronic inflammatory disease of the nasal cavity and paranasal sinuses, and represents the most frequent type of fungal rhinosinusitis. In recent years, epidemiological studies from multiple research centers have shown that the incidence of FBS presents a significant upward trend (1, 2), imposing a heavy burden on clinical medical resources. Currently, FBS is considered to be associated with long-term use of antibiotics, poor blood glucose control, immunosuppressants, or radiotherapy (3). FBS has distinct demographic characteristics, with a higher incidence in middle-aged and elderly populations, and the gender difference in FBS incidence has attracted increasing attention, with a significantly higher incidence in women than in men (2).

Anatomical abnormalities of the nasal cavity and paranasal sinuses can lead to poor ventilation and drainage, forming a humid, closed sinus environment that is conducive to fungal colonization and proliferation, which is a common comorbidity of FBS (2). In addition, chronic diseases such as diabetes mellitus, nephropathy, and tumor history are also important risk factors for FBS (3). Most of these patients have immune insufficiency or metabolic disorders, which further reduce the body's resistance to fungi. Studies have suggested that immune responses exhibit significant gender differences (4, 5). The mechanisms underlying the female predominance in FBS remain unclear. Studies have indicated that metabolic factors such as body mass index (BMI) affect the occurrence of various inflammatory diseases, including chronic rhinosinusitis with nasal polyps, allergic rhinitis, and asthma (6–8). However, perimenopausal women, a high-risk group, are often accompanied by metabolic disorders such as insulin resistance and dyslipidemia, which may further increase the risk of FBS, but the specific association has not been clarified.

High-density lipoprotein cholesterol (HDL-C), also known as “good cholesterol”, is an important indicator of lipid metabolism, which is mainly responsible for reverse cholesterol transport, anti-oxidation, anti-inflammation, and protection of vascular endothelium (9). Decreased HDL-C level usually indicates a lipid metabolism disorder and reduced anti-inflammatory capacity of the body. Whether the disorder of HDL-C is related to the pathogenesis of FBS deserves further exploration. Triglyceride-glucose index (TyG) is a simple and practical indicator for evaluating insulin resistance, and TyG-BMI index further integrates the dual effects of obesity and insulin resistance (10). Insulin resistance can affect the function of immune cells and promote the release of systemic inflammatory factors (11–13), which may aggravate chronic inflammation of the nasal and paranasal sinus mucosa and change the sinus microenvironment, facilitating fungal growth and reproduction. Based on the fact that diabetic patients with poor blood glucose control are high-risk groups for FBS, whether insulin resistance increases the risk of FBS remains unclear. The correlation between TyG-BMI, HDL-C and FBS in perimenopausal women has not been reported.

Based on the research background, this study retrospectively analyzed the epidemiological characteristics of FBS patients, focusing on the gender and age differences of FBS. Then, taking perimenopausal women (45–60 years old) as the research objects, the differences in metabolic and blood routine indicators between the FBS group and chronic non-fungal rhinosinusitis (CNFS) group were analyzed. Independent risk factors of FBS in perimenopausal women were determined by logistic regression analysis. Finally, the ROC curve was used to analyze the predictive value of HDL-C and TyG-BMI for FBS. This study aims to provide a new theoretical basis and clinical reference for the prevention, early identification, and intervention of FBS in perimenopausal women.

## 2 Patients and methods

### 2.1 Data collection

This study used a retrospective cohort design to include patients diagnosed with FBS at our hospital between 2015 and 2025. Patients who underwent endoscopic sinus surgery and were pathologically diagnosed as FBS were included in the subsequent analysis. Clinical data of all patients were collected, including gender, age, height, weight, clinical diagnoses, as well as blood routine and biochemical routine indicators. This study was approved by the Medical Ethics Committee of the Affiliated Hospital of Southwest Medical University (KY2026252). The requirement for informed consent was waived by the ethics committee due to the retrospective and anonymized nature of the study.

### 2.2 The characteristics of FBS in perimenopausal women

Inclusion criteria: Female patients aged 45–60 years (the age range of perimenopausal women) with complete preoperative clinical, laboratory, and imaging data. Patients with missing preoperative biochemical or blood routine data were excluded from the analysis. Approximately 16.9% of screened patients were excluded due to missing laboratory data.

Exclusion criteria: Patients with any of the following conditions were excluded to eliminate confounding factors: 1) History of allergic rhinitis (AR) or nasal polyps; 2) Diagnosis of diabetes mellitus (type 1 or 2); 3) Immunodeficiency diseases or long-term use of systemic glucocorticoids/immunosuppressants; 4) History of malignant tumor; 5) Hepatic dysfunction (transaminase elevation > 2 times the upper limit of normal); 6) History of sinus surgery.

Female patients aged 45–60 years with non-fungal chronic rhinosinusitis (CNFS) hospitalized in the same period were selected as the control group, with the same exclusion criteria as above. All biochemical indicators including total cholesterol (TC), triglyceride (TG, mmol/L), high-density lipoprotein cholesterol (HDL-C, mmol/L), low-density lipoprotein cholesterol (LDL-C, mmol/L), fasting blood glucose (Glu, mmol/L), as well as blood routine indicators including white blood cells (WBC), eosinophils (EOS) and eosinophil ratio (EOS%) were detected from preoperative fasting blood samples by the hospital's laboratory department. BMI = weight (kg)/height (m)^2^; TyG = ln[TG[mg/dL] × Glu[mg/dL]/2]; TyG-BMI index = TyG × BMI. TG and Glu in the TyG formula are in mg/dL. If original laboratory data are in mmol/L, conversion is required: TG (mg/dL) = TG (mmol/L) × 88.57; Glu (mg/dL) = Glu (mmol/L) × 18.02.

### 2.3 Statistical analysis

Statistical analysis was performed using GraphPad Prism 9 (San Diego, CA, USA) and IBM SPSS Statistics 26.0 (IBM, Armonk, NY, USA). The Shapiro–Wilk test was applied to assess the normality of continuous variables. Normally distributed continuous data were presented as mean ± standard deviation (SD) and compared by independent-samples *t*-test; non-normally distributed continuous data were presented as median (interquartile range, IQR) and compared by the Mann–Whitney *U*-test (two groups) or Kruskal–Wallis *H*-test (multiple groups). Categorical variables were reported as counts and percentages, and compared using the *χ*^2^-test.

Logistic regression was performed to identify risk factors for FBS. Continuous TyG-BMI, TyG and BMI were excluded from the final multivariate model to avoid multicollinearity and redundant information. The predictive performance was evaluated via the area under the receiver operating characteristic curve (AUC). *P* < 0.05 was considered statistically significant.

## 3 Results

### 3.1 Clinical characteristics of FBS

Between 2015 and 2025, 826 patients with high suspicion of fungal ball sinusitis based on CT imaging features but without surgical and pathological confirmation were hospitalized in our hospital. A total of 1,440 patients who underwent endoscopic sinus surgery and were pathologically confirmed as FBS in our department were enrolled, including 1,205 (83.68%) cases of maxillary sinus fungal ball, 232 (16.11%) cases of sphenoid sinus fungal ball, and 26 (1.8%) cases of concurrent nasal septum deviation correction. Overall, the number of FBS showed a significant upward trend over the year (Figure 1A). The mean age of patients was 54.84 ± 12.6 years, mainly middle-aged and elderly patients. In terms of comorbidities, there were 160 (11.11%) cases complicated with diabetes mellitus, 11 (0.8%) cases with malignant tumors, and 187 (12.99%) cases with AR. Among all FBS patients, 921 (63.96%) were female, and 519 (36.04%) were male, with a female: male ratio of 1.77:1. The proportion of females was significantly higher than that of males (*P* < 0.05), and the increasing trend in females was more significant than that in males (Figure 1A). Age distribution analysis showed that the peak age of FBS onset in females was 45–60 years, accounting for 55% of all female FBS patients (Figure 1B). The violin plot further illustrates age distributions of FBS patients stratified by sex. Statistical comparisons revealed significant age-related differences among female FBS patients (*P* < 0.001), whereas no significant age difference was observed in male FBS patients (*P* = 0.3651) (Figure 1B).

**Figure 1:**
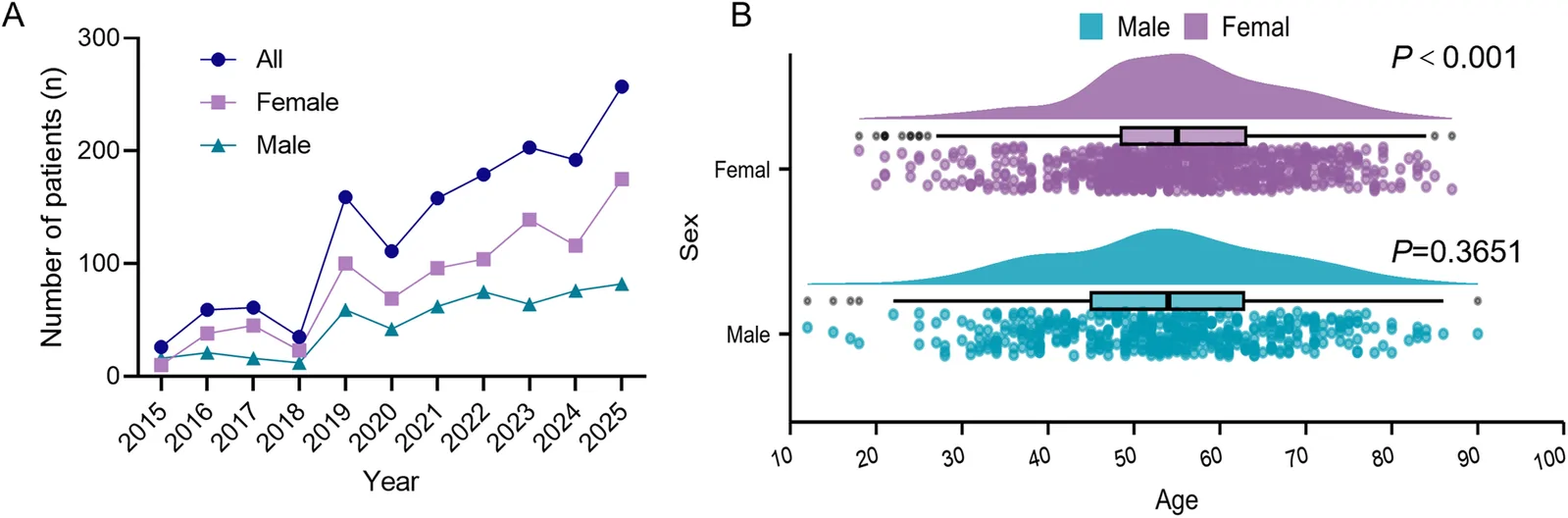
The temporal and age trends of FBS incidence. **(A)** Temporal trend of FBS in different genders; stratified by sex. green line: male; purple line: female; blue line: total **(B)** Age distribution of FBS patients grouped by sex. The peak age group is 45-60 years, accounting for 55% of all female FBS patients.

### 3.2 Study population characteristics of FBS in perimenopausal women

According to the inclusion/exclusion criteria, a total of 264 female FBS patients aged 45–60 years and 266 CNFS patients were enrolled in this study. The baseline characteristics of the two groups are summarized in Table 1. Age was well-matched between the FBS and CNFS groups (52.50 vs. 53.00 years, *P* = 0.779). TG, BMI, TyG, and TyG-BMI were significantly higher, and HDL-C was significantly lower in the FBS group than in the CNFS group (all *P* < 0.05). No significant differences were observed in TC, LDL-C, Glu, WBC, EOS, or EOS% between the two groups (all *P* > 0.05). The biochemical and blood routine indicators between the two groups were compared. The results showed that HDL-C in the FBS group was significantly lower than that in the CNFS group (*P* = 0.0002, Figure 2A). In contrast, TyG and TyG-BMI in the FBS group were significantly higher than those in the CNFS group (*P* = 0.0024 and 0.0094, Figures 2D,E), and the TyG-BMI index was significantly negatively correlated with HDL-C (*P* < 0.0001, *R* = −0.3936, Figure 2F). However, there were no statistically significant differences in LDL-C, BMI, EOS, and EOS% between the two groups (Figures 2B,C).

**Table 1:** Baseline characteristics of FBS and CNFS perimenopausal women.

Variable	FBS (*n* = 264)	CNFS (*n* = 266)	P
Age (years)	52.50 (7.00)	53.00 (7.00)	0.779
TC (mmol/L)	5.09 (1.28)	5.05 (1.33)	0.818
TG (mmol/L)	1.36 (0.98)	1.16 (0.88)	<0.001
HDL-C (mmol/L)	1.48 (0.43)	1.56 (0.50)	0.001
LDL-C (mmol/L)	3.18 (1.02)	3.04 (1.06)	0.616
Glu (mmol/L)	5.16 (0.67)	5.20 (0.70)	0.567
WBC (×10^9^/L)	5.62 (1.90)	5.60 (1.77)	0.679
EOS (×10^9^/L)	0.09 (0.11)	0.09 (0.12)	0.923
EOS%	1.70 (1.80)	1.60 (1.80)	0.630
BMI (kg/m^2^)	24.34 (3.91)	23.83 (4.15)	0.020
TyG	8.63 (0.73)	8.46 (0.71)	<0.001
TyG-BMI	208.68 (43.60)	201.68 (41.43)	0.003

Data are presented as median (IQR). *P* values were calculated using the Mann–Whitney *U*-test.

**Figure 2:**
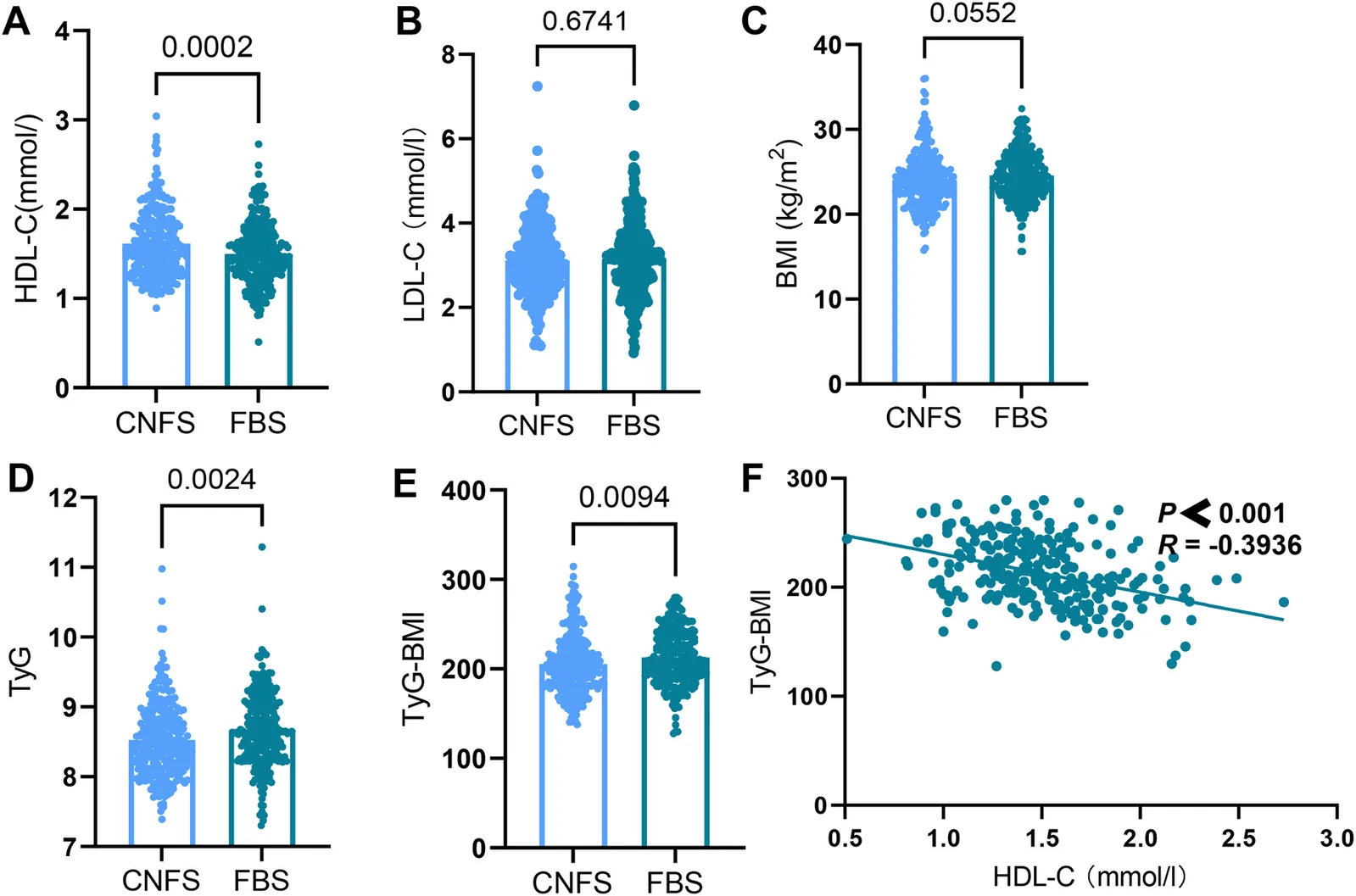
**(A–E)** differential analysis of metabolism-related indicators between FBS and CNFS groups. Differences in HDL-C **(A)**, LDL-C **(B)**, BMI **(C)**, TyG **(D)**, and TyG-BMI **(E)** between FBS and CNFS groups. **(F)** Correlation between TyG-BMI index and HDL-C in perimenopausal women (*n* = 530). Spearman correlation coefficient R = −0.3936, *P* < 0.001.

### 3.3 Logistic analysis of tyG-BMI and HDL-C with FBS

The patients with FBS and CNFS were divided into four groups (Q1, Q2, Q3, Q4) according to TyG-BMI quartiles, and the baseline clinical and biochemical indicators among the four groups were compared (Table 2). The results showed no statistically significant differences in age and EOS% among the four groups (*P* > 0.05); with the increase of TyG-BMI quartiles, TC, TG, BMI, LDL-C, and TyG gradually increased, while HDL-C level gradually decreased, with statistically significant overall differences among groups (*P* < 0.05). Meanwhile, with the increase in TyG-BMI quartile level, the prevalence of FBS gradually increased. The risk of FBS onset showed a significant upward trend with the increase of TyG-BMI, with statistically significant trend differences among groups (*P* < 0.05) (Table 2).

**Table 2:** Comparison of baseline data among different tyG-BMI quartile groups.

Variable	Q1 (*n* = 133)	Q2 (*n* = 132)	Q3 (*n* = 132)	Q4 (*n* = 133)	*P*
Age (years)	53.068	51.826	52.621	52.301	0.1
TC (mmol/L)	5.1068	4.993	5.207	5.448	0.003
TG (mmol/L)	1.008	1.236	1.638	2.314	**< 0.001**
HDL-C (mmol/L)	1.783	1.564	1.485	1.395	**< 0.001**
LDL-C (mmol/L)	2.992	3.002	3.202	3.333	0.001
Glu (mmol/L)	5.084	5.125	5.219	5.293	0.015
WBC (×10^9^/L)	5.824	5.739	6.179	6.270	0.018
EOS (×10^9^/L)	0.135	0.119	0.145	0.147	0.025
EOS%	2.129	2.110	2.306	2.427	0.241
BMI (kg/m^2^)	20.855	23.385	24.725	28.166	**< 0.001**
TyG (mmol/L)	8.230	8.435	8.744	8.982	**< 0.001**
FBS (*n*, %)	49 (18.6%)	69 (26.1%)	68 (25.8%)	78 (29.5%)	0.001

Bold values indicate statistically significant *P* values (*P* < 0.05).

Univariate logistic regression analysis was performed to identify potential risk factors for FBS in perimenopausal women (Figure 3). The results showed that HDL-C, TyG-BMI, quartile of TyG-BMI (Q of TyG-BMI), and TyG were significantly associated with FBS risk (*P* < 0.05). Specifically, HDL-C was inversely associated with FBS (OR = 0.391, 95%CI: 0.238–0.644, *P* < 0.001), indicating a protective effect. TyG-BMI (OR = 1.006, 95%CI: 1.001–1.012, *P* = 0.019), Q of TyG-BMI (OR = 1.299, 95%CI: 1.113–1.517, *P* = 0.001), and TyG (OR = 1.661, 95%CI: 1.191–2.316, *P* = 0.003) were positively associated with increased FBS risk. No significant associations were found for age, TC, TG, LDL-C, Glu, EOS, EOS%, or BMI (*P* > 0.05).

**Figure 3:**
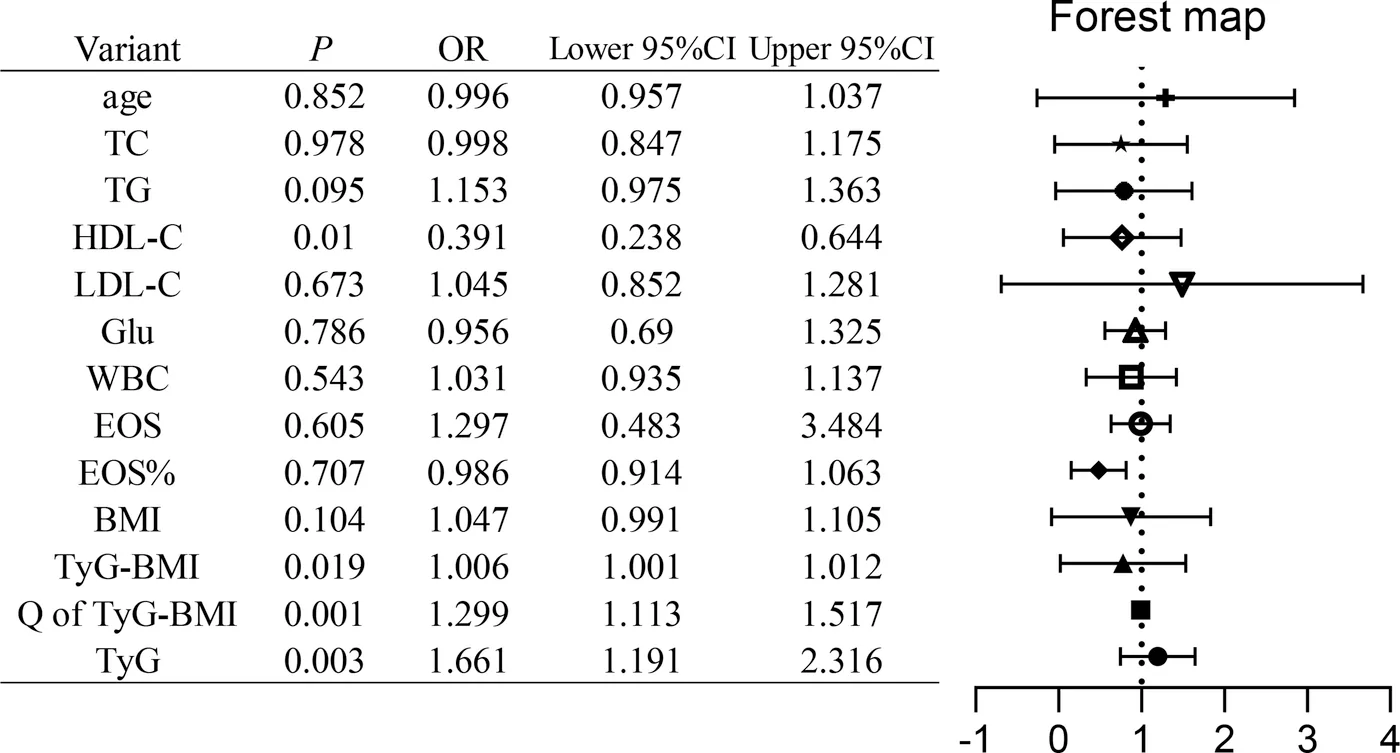
Univariate logistic regression analysis of FBS in perimenopausal women.

The multivariate logistic regression model, adjusted for Q of TyG-BMI and HDL-C, showed that Q of TyG-BMI was an independent risk factor for FBS (OR = 1.192, 95%CI: 1.008–1.409, *P* = 0.04). HDL-C was an independent protective factor for FBS (OR = 0.482, 95%CI:0.282–0.823, *P* = 0.008). The detailed results are shown in Table 3.

**Table 3:** Multivariate logistic regression analysis of FBS in perimenopausal women.

Variant	B	SE	Wald *χ*²	*P*	OR	95%CI	
						Lower	Upper
Q of TyG-BMI	0.175	0.085	4.232	0.04	1.192	1.008	1.409
HDL-C	−0.73	0.273	7.137	0.008	0.482	0.282	0.823

### 3.4 ROC curve analysis

ROC curve was used to analyze the predictive value of HDL-C, TyG-BMI quartiles, and TyG-BMI index for FBS in perimenopausal women. The results showed that none of them had good predictive value, and the combined predictive ability was improved to a certain extent, with an AUC of 0.618 (95% CI: 0.571–0.665, *P* < 0.05) (Figure 4).

**Figure 4:**
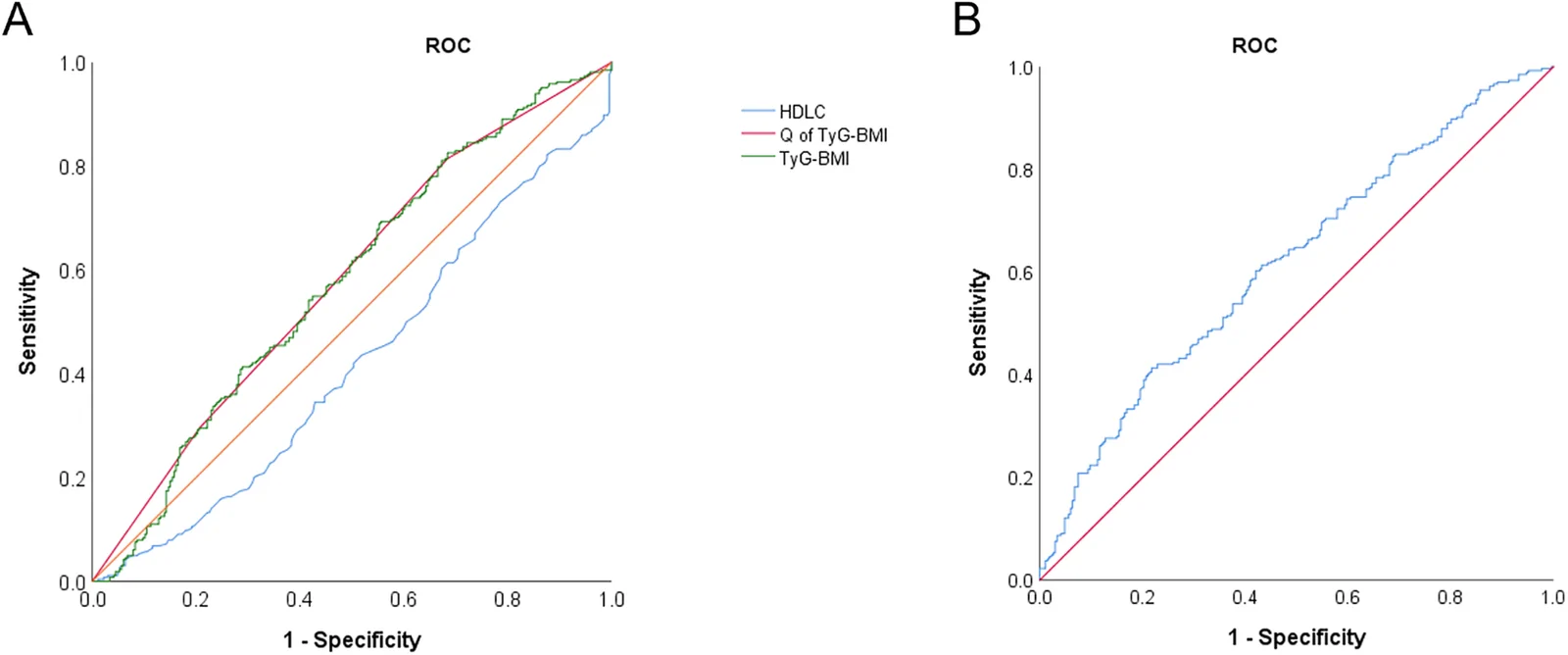
Diagnostic value of metabolism-related indicators in FBS. **(A)** ROC curves for HDL-C, Q of TyG-BMI, and TyG-BMI. **(B)** ROC curve of the combined model. The combined model yielded an AUC of 0.618 (95%CI: 0.571–0.665, *P* < 0.05).

## 4 Discussion

In 2009, the International Society for Human and Animal Myology classified fungal rhinosinusitis (FRS) into invasive and non-invasive types according to histopathological results. The latter includes fungal ball sinusitis (FBS) and allergic fungal rhinosinusitis (AFRS) (14). FBS is characterized by the formation of fungal masses in the sinuses, with insidious onset and a serious impact on patients' quality of life (14). This study found that the number of FBS surgical cases showed a significant upward trend during 2015–2025, which is consistent with reports of increasing FBS incidence worldwide (1, 2). Potential reasons include the aging population, environmental factors (such as air pollution and allergen exposure), irrational use of antibiotics and hormones, as well as the popularization and improvement of clinical diagnostic techniques (such as nasal endoscopy and imaging examination), leading to the early detection and surgical treatment of more insidious FBS cases (3, 15). FBS mainly occurs in middle-aged and elderly people. Notably, the proportion of female FBS patients in this study was significantly higher than that of males, which is consistent with the results of retrospective analyses from other centers (1, 2, 16). The peak onset age in females is 45–60 years, suggesting that perimenopausal women may be a high-risk group for FBS. Studies have suggested that gender differences may be the intrinsic pathological and recurrence factors of chronic rhinosinusitis with nasal polyps (17). The potential mechanism underlying the gender differences in this study may be closely related to endocrine disorders in perimenopausal women: the level of estrogen in perimenopausal women decreases significantly, and estrogen and progesterone can affect the density of muscarinic and *α*1-adrenergic nasal mucosal receptors (18). In addition, perimenopausal women are often accompanied by mood swings, sleep disorders, and increased sensitivity, which further weaken the body's overall immunity and create conditions for fungal colonization (19–21).

This study focused on perimenopausal women aged 45–60 years. The HDL-C level in the FBS group was significantly lower than that in the CNFS group, and the TyG and TyG-BMI levels were significantly higher than those in the CNFS group, suggesting that metabolic disorders may be involved in the pathogenesis of FBS in perimenopausal women. As a “good cholesterol”, HDL-C is mainly responsible for reverse cholesterol transport, anti-oxidation, anti-inflammation, and protection of vascular endothelium. Its decrease usually indicates lipid metabolism disorder and reduced anti-inflammatory capacity of the body (9, 22). Previous studies have shown that decreased HDL-C levels may be related to colonic mucosal inflammation and impaired mucosal barrier function in mice (23). TyG index is a simple indicator reflecting insulin resistance, and TyG-BMI combined with BMI further integrates the dual effects of obesity and insulin resistance. The increase in both indicates obvious metabolic abnormalities in the body (10, 24). Poor blood glucose control is an important factor in the pathogenesis of fungal rhinosinusitis. Insulin resistance can affect the function of immune cells, promote the release of inflammatory factors (25, 26), aggravate chronic inflammation of the nasal and paranasal sinus mucosa, and change the microenvironment in the sinuses, which is conducive to the growth and reproduction of fungi. Further exploring the dose-response relationship between TyG-BMI quartiles and FBS showed that the higher the TyG-BMI level, the higher the risk of FBS in perimenopausal women. This result further demonstrates a close correlation between metabolic disorders and FBS. Recent studies have suggested that BMI is related to the pathological intrinsic type, recurrence, and therapeutic effect of biological agents in chronic rhinosinusitis with nasal polyps. However, there was no statistical difference in BMI between the two groups in this study (7, 27–29). The exclusion of patients with diabetes mellitus may partly account for the absence of a significant between-group difference in BMI. Given the strong association between diabetes and obesity-related metabolic dysfunction, excluding diabetic patients may have shifted the study population toward a metabolically healthier profile, thereby attenuating the detectable difference in BMI between the FBS and CNFS groups.

This study revealed that low HDL-C and high TyG-BMI are independent risk factors for FBS in perimenopausal women, while TyG is not. This is because TyG-BMI, as a composite index integrating insulin resistance and obesity, can more comprehensively reflect the metabolic status of the body (30, 31). As an independent protective factor, the decrease in HDL-C level significantly increases the risk of FBS, suggesting that higher HDL-C levels were associated with a lower risk of FBS in perimenopausal women. Unfortunately, the combined model of TyG-BMI and HDL-C yielded an AUC of 0.618, indicating limited predictive value. This may be attributed to the complex and multifactorial pathogenesis of FBS, which cannot be fully captured by metabolic indicators alone. Other potential predictors, such as anatomical abnormalities, environmental exposure, and local immune status, were not included. Additionally, the relatively small sample size may affect the stability of the prediction model. Future studies should incorporate imaging and immune markers to improve predictive performance.

## 5 Limitations and future directions

This study has certain limitations. Firstly, as a retrospective analysis, there is selection bias, and some clinical data may be missing, affecting the accuracy of the results. In future studies, prospective studies can be carried out with expanded sample size to reduce selection bias. Secondly, the specific molecular mechanism of TyG-BMI and HDL-C affecting FBS in perimenopausal women was not deeply explored, which needs to be further studied in basic research to clarify the association between metabolic disorders, fungal colonization and mucosal inflammation. Thirdly, the control group consisted of hospitalized patients with CNFS rather than population-based healthy subjects. These patients may also harbor underlying metabolic abnormalities, which may attenuate the true effect size of metabolic markers for FBS. Incorporating healthy controls in future studies would help validate these findings. Fourthly, perimenopausal status was defined by age 45–60 years rather than by hormonal or menstrual-cycle criteria. This approach is consistent with international clinical guidelines, which do not recommend hormonal testing for identifying perimenopause in women aged 45 years or older, as clinical assessment and age are considered sufficient for this purpose (32). Nevertheless, an age-based definition may introduce misclassification bias, as some women within this range may still be premenopausal or already postmenopausal. Hormonal profiles and menstrual history should be used to confirm perimenopausal status in future studies. Finally, the efficacy of the combined prediction model needs to be improved. In the future, the prediction model can be further optimized by combining imaging indicators and immune indicators to improve the early prediction and intervention ability of FBS in perimenopausal women.

## 6 Conclusion

The incidence of FBS is rising, particularly among perimenopausal women. Low HDL-C and elevated TyG-BMI are independent risk factors for FBS. These modifiable metabolic factors highlight the need for monitoring and early intervention in high-risk perimenopausal women to potentially reduce FBS risk.

## Data Availability

The original contributions presented in the study are included in the article/Supplementary Material, further inquiries can be directed to the corresponding author/s.
